# Total Hip Arthroplasty for Osteonecrosis of the Femoral Head Following Allogeneic Haematopoietic Stem Cell Transplantation, with Graft-Versus-Host Disease as a Candidate Effect Modifier: A Systematic Review and Meta-Analysis

**DOI:** 10.3390/jcm15197528

**Published:** 2026-09-28

**Authors:** Minki Beak, Young Wook Lim

**Affiliations:** 1 Department of Community Health, Tufts University, Medford, MA 02155, USA; minki.beak@tufts.edu; 2 Department of Orthopaedic Surgery, Seoul St. Mary’s Hospital, College of Medicine, The Catholic University of Korea, 222 Banpo-daero, Seocho-gu, Seoul 06591, Republic of Korea

**Keywords:** total hip arthroplasty, osteonecrosis of the femoral head, haematopoietic stem cell transplantation, graft-versus-host disease, periprosthetic joint infection, implant survivorship

## Abstract

**Background/Objectives:** Osteonecrosis of the femoral head is a common late complication of allogeneic haematopoietic stem cell transplantation, driven largely by graft-versus-host disease (GVHD) and its immunosuppressive treatment. We evaluated implant survivorship, all-cause revision and periprosthetic joint infection or septic loosening after total hip arthroplasty, and whether GVHD modifies these outcomes. **Methods:** Six databases were searched from inception to July 2026. The review was registered with PROSPERO (CRD420261446131) and reported per PRISMA 2020. Proportions were pooled with a random-intercept logistic model, with sensitivity analyses for the registered Freeman–Tukey approach, follow-up duration, within-patient clustering and competing mortality. Risk of bias used ROBINS-I and MINORS, certainty GRADE; GVHD was the prespecified effect modifier. **Results:** Six studies were included; four cohorts (228 hips) were pooled. Pooled all-cause revision was 4.39% (95% CI 2.38–7.96) and pooled infection or septic loosening 1.05% (95% CI 0.28–3.87). In the two cohorts followed beyond five years (182 hips), these rose to 5.49% (95% CI 2.98–9.91) and 1.62% (95% CI 0.51–5.01), the two shorter cohorts recording no event in 46 hips. All three infection events arose in one cohort, in patients with chronic GVHD on steroids at revision. With no GVHD-stratified denominators reported, however, this remains descriptive, not an estimable relative risk. Certainty was very low throughout. **Conclusions:** Total hip arthroplasty after allogeneic transplantation is durable over the first post-operative decade. All infection-related failures arose in patients with chronic GVHD on continuing immunosuppression—a mechanistically plausible pattern, but one resting on three events in one cohort and still unquantified. GVHD-stratified reporting is the next step.

## 1. Introduction

Osteonecrosis of the femoral head is a leading indication for total hip arthroplasty and, unlike primary osteoarthritis, characteristically affects younger patients, for whom implant longevity and lifetime revision risk matter disproportionately [1,2]. A distinct form of osteonecrosis follows allogeneic haematopoietic stem cell transplantation. Acute graft-versus-host disease (GVHD) develops in roughly 30–70% of recipients and chronic GVHD in 27–50% of long-term survivors of sibling-donor grafts, with higher rates after unrelated-donor and peripheral blood stem cell transplantation [3,4]. GVHD is treated with prolonged high-dose corticosteroids, frequently with additional systemic immunosuppression, and this treatment pattern is the dominant risk factor for osteonecrosis in transplant recipients [5,6]. In a cohort of 902 consecutive bone marrow transplants, osteonecrosis developed in 28 of 642 allogeneic recipients (10.4% by product-limit estimate) but in none of 260 autologous recipients, and GVHD requiring steroid therapy was a significant risk factor [7]. Osteonecrosis affects roughly 3–10% of adult allogeneic recipients within five years [8,9] and a higher proportion of children [10]. The sequence of transplantation, GVHD, immunosuppression, osteonecrosis and arthroplasty defines the population of interest. Cohort studies suggest that modern arthroplasty performs well here, with survivorship approaching that of osteonecrosis from other causes [2,11]. Yet in the largest series all septic loosening occurred in patients with chronic GVHD on continuing steroids [1], raising the question of whether GVHD and its treatment shift the failure mode towards infection rather than raising failure overall. Existing syntheses have pooled osteonecrosis broadly or compared it with osteoarthritis without isolating the transplant subgroup, and neither has treated GVHD as the explanatory variable [12,13]. We therefore systematically reviewed implant survivorship, all-cause revision and infection-related failure after arthroplasty for osteonecrosis following allogeneic transplantation and evaluated whether GVHD and its immunosuppressive treatment modify these outcomes.

## 2. Materials and Methods

### 2.1. Protocol and Registration

This review is reported per the PRISMA 2020 statement [14] and conducted per a protocol registered with PROSPERO (CRD420261446131). Preliminary scoping searches were undertaken to confirm the feasibility and scope of the review before registration; the formal searches and screening reported here followed registration. This study analysed previously published aggregate data, involved no human participants or animals, and required neither ethics committee approval nor informed consent.

### 2.2. Eligibility Criteria

We included comparative cohorts and case series reporting primary total hip arthroplasty for osteonecrosis of the femoral head after allogeneic transplantation, with at least ten hips and a mean follow-up of at least 24 months. GVHD, acute or chronic, together with ongoing corticosteroid or immunosuppressive therapy, was the exposure of interest. Primary outcomes were periprosthetic joint infection or septic loosening, revision, and Kaplan–Meier survivorship. Secondary outcomes were aseptic loosening, functional scores, complications and mortality. Reviews, case reports and abstracts without extractable data were excluded, as were studies in which the post-transplant hip subgroup could not be identified separately. Where cohorts overlapped, the report with the longest follow-up was retained. The full criteria are given in Table S1.

### 2.3. Information Sources and Search

PubMed, MEDLINE (Ovid), Embase, Cochrane CENTRAL, Web of Science and Scopus were searched from inception to July 2026, and supplemented with reference-list screening which yielded no additional eligible reports. No trial register was searched separately, but two ClinicalTrials.gov records were retrieved within the Embase results and are counted in the Embase total. The search strategy combined four concept blocks: transplantation, GVHD or immunosuppression, osteonecrosis, and arthroplasty. The search combined controlled vocabulary with free-text terms and was limited to English. The full syntax is given in Table S2.

### 2.4. Selection and Data Extraction

Records were de-duplicated in reference-management software (Zotero, version 10.0; Corporation for Digital Scholarship, Vienna, VA, USA). Two reviewers independently screened titles, abstracts and full texts and independently extracted study, population, exposure, implant and outcome data, resolving disagreements by discussion until consensus was reached. Consensus was reached in every instance and no disagreement required adjudication beyond the two reviewers. Every report was read in full and denominators and event counts were taken from operative and reoperation tables in preference to summary statements, and disagreements over such factual items were settled by returning together to the primary report.

### 2.5. Risk of Bias and Certainty

Non-randomised comparative studies were appraised with ROBINS-I across its seven domains, the overall judgement taken from the least favourable domain [15]. Non-comparative series were assessed with MINORS, which scores each of eight items from 0 to 2 for a maximum of 16 [16]. No randomised trials were identified. Particular attention was paid to confounding by indication and GVHD severity. Two reviewers assessed the studies independently and resolved disagreements by discussion; no study was excluded on quality alone. Certainty of the pooled evidence was rated using the GRADE approach [17].

### 2.6. Statistical Synthesis

Proportions were pooled with a random-intercept logistic model (binomial–normal generalised linear mixed model) fitted by adaptive Gauss–Hermite quadrature, which models the binomial likelihood directly, requires no continuity correction and admits zero-event cohorts. Only cohorts comprising exclusively allogeneic recipients undergoing arthroplasty for osteonecrosis were pooled. Two studies in which the post-allogeneic osteonecrosis subgroup could not be separated were synthesised narratively, consistent with the registered protocol. Denominators were the number of hips implanted rather than the number under review at last contact, because restricting the analysis to hips reviewed at final follow-up would condition on survival and discard the hips of patients who had died. The registered protocol had specified a DerSimonian–Laird random-effects model [18] on Freeman–Tukey double-arcsine transformed data [19]. That approach was retained as a prespecified sensitivity analysis but demoted from the primary analysis because, for the infection outcome, the inverse transformation returned a point estimate of 0.49%, well below the crude rate of 1.32%, which is a documented instability of this back-transformation at low proportions and with unequal cohort sizes [20]. The deviation is declared in Section 4.5: Limitations. Exact (Clopper–Pearson) intervals on the aggregated counts are reported as a second, purely descriptive sensitivity analysis. Between-study heterogeneity was summarised by τ^2^ from the mixed model and, because Q and I^2^ are scale-dependent, by Cochran’s Q and I^2^ computed on the double-arcsine scale [21]. Survivorship reported at differing landmarks and against differing endpoints was summarised narratively rather than pooled quantitatively. GVHD was the prespecified effect modifier and a relative-risk meta-analysis was contingent on GVHD-stratified denominators. Three further sensitivity analyses were added at revision. The first stratified the estimates by mean follow-up above and below five years, the interval in which septic loosening was observed to occur. The second was a cluster-robust recomputation of the confidence intervals using a design effect of 1 + ($\bar{m}$ − 1)ρ, with $\bar{m}$ = 1.689 hips per patient and ρ = 1 as the worst case of complete within-patient correlation. The third was a survivor-conditional analysis excluding the hips of patients who died during follow-up. All sensitivity estimates for both outcomes are reported together in Table S6. Funnel plots and Egger’s test were not performed because fewer than ten studies were available. Analyses were performed in R version 4.6.1 (R Foundation for Statistical Computing, Vienna, Austria) using the meta (version 8.5-0) and metafor (version 5.0-1) packages [22].

## 3. Results

### 3.1. Study Selection

The search returned 150 records. After removing 62 duplicates, 88 records were screened and 77 were excluded. Eleven full texts were assessed, of which five were excluded and six met the eligibility criteria (Figure 1). Two were earlier, superseded reports of a cohort whose later and larger series was retained [23,24]. One reported hip arthroplasty in only two of five affected patients, below the prespecified minimum of ten hips [25]. One described the incidence, clinical course and risk factors of avascular necrosis after transplantation without reporting arthroplasty outcomes [7]. One studied corticosteroid-induced osteonecrosis after treatment for haematological malignancy, in which only four of 27 patients had undergone transplantation and the post-transplant subgroup was not reported separately [26]. Four cohorts comprising exclusively allogeneic recipients undergoing arthroplasty for osteonecrosis, totalling 228 hips, provided extractable event counts and entered the quantitative synthesis [1,2,27,28]. Two further studies were synthesised narratively. One study is an institutional registry cohort in which allogeneic and autologous recipients could not be separated and only 23 of 42 hips were for osteonecrosis [29], and the other study reported combined hip and knee arthroplasty with combined indications [30].

### 3.2. Study Characteristics

The cohorts were published between 2008 and 2024 in France, South Korea, Greece and the United States, and all were retrospective studies (Table 1). Mean follow-up ranged from 44.5 months to 10.6 years. GVHD burden was high throughout, affecting most patients in every cohort [1,2,29] and all patients in two [27,28], though only one study reported the affected proportion as an explicit numerator and denominator [2].

### 3.3. Risk of Bias

Every included study carried an important methodological limitation (Tables S3 and S4). Across the two comparative cohorts, confounding was the domain of primary concern: neither study adjusted, matched or stratified for GVHD status or cumulative corticosteroid exposure, so confounding by GVHD status or immunosuppressive therapy cannot be excluded. The propensity-matched cohort was matched on age, sex, body mass index, physical status and year of surgery, but not on any GVHD-related variable [2]. The other domains performed well, however. Classification of intervention was at low risk throughout the review, and in the registry-based and propensity-matched cohorts the ascertainment of revision and infection (using the Musculoskeletal Infection Society criteria) was also at low risk, lending credibility to the low pooled event rates. The four non-comparative series scored 6 to 12 of a maximum of 16 on MINORS, three at moderate and one at high risk of bias, with consistent deductions based on factors such as retrospective collection, unblinded assessment and absent sample-size calculation.

### 3.4. Revision and Infection

Study-level outcomes are given in Table 2. Across the four cohorts (228 hips, 10 revisions), pooled all-cause revision was 4.39% (95% CI 2.38–7.96), with negligible between-study variance (τ^2^ < 0.001 on the logit scale; I^2^ = 15%, Q = 3.52, df = 3, *p* = 0.32 on the double-arcsine scale) (Figure 2). Pooled periprosthetic joint infection or septic loosening (three events) was 1.05% (95% CI 0.28–3.87; τ^2^ = 0.30 on the logit scale; I^2^ = 0%, Q = 2.81, df = 3, *p* = 0.42), the between-study variance being poorly identified because three of the four cohorts reported no events (Figure 3). The model-based revision estimate coincides with the crude rate (10 of 228), indicating that no single cohort dominates the pooled figure. In sensitivity analyses the prespecified Freeman–Tukey model gave 3.20% (95% CI 0.79–6.70) for revision and, for infection, a point estimate of 0.49%, well below the crude rate of 1.32%, illustrating the known instability of that back-transformation with sparse events [20]. The exact binomial intervals on the aggregated counts gave 4.39% (95% CI 2.12–7.92) and 1.32% (95% CI 0.27–3.80) respectively. Recomputing the intervals under the worst-case assumption of complete within-patient correlation (design effect 1.689, 228 hips from 135 patients) widened them to 1.97–9.46 for revision and 0.19–5.66 for infection without moving either point estimate, and restricting the denominator to the 201 hips of patients alive at last follow-up gave 4.98% (95% CI 2.70–9.00) and 1.09% (95% CI 0.26–4.42). All sensitivity estimates for both outcomes are collected in Table S6.

Because septic loosening in this literature declared itself between 6.5 and 9.4 years after surgery, the pooled estimates were stratified by mean follow-up, the sensitivity analysis advised in Table S4. The two cohorts followed beyond five years (182 hips) gave all-cause revision of 5.49% (95% CI 2.98–9.91) and periprosthetic joint infection or septic loosening of 1.62% (95% CI 0.51–5.01), whereas the two cohorts followed for means of 3.7 and 4.7 years recorded no event of either kind in 46 hips (0%, 95% CI 0–7.71). Every observed event therefore falls in the stratum long enough to observe it, and the whole-cohort figures sit below those of that stratum. The pooled infection estimate of 1.05% is accordingly best read as a lower bound across cohorts of unequal observation time rather than as an overall infection rate.

### 3.5. Secondary Outcomes

Four secondary outcome domains were prespecified. Only all-cause mortality could be synthesised quantitatively and the remainder are described narratively.

#### 3.5.1. Aseptic Loosening

Aseptic loosening was prespecified separately from septic loosening but could not be pooled, because the four cohorts did not measure the same thing. The largest cohort defined loosening quantitatively, as cup migration exceeding 3 mm, rotation exceeding 3° or a radiolucent line wider than 2 mm [1]. One series reported no loosening at a mean of 56.4 months using Engh criteria with DeLee–Charnley and Gruen zones [27]. A second reported no failures at a mean of 44.5 months but nominated no loosening threshold, no radiographic zone system and no method of wear measurement, so its null result cannot be read as a measured absence of loosening [28]. The propensity-matched cohort reported revision rather than loosening [2].

#### 3.5.2. Functional and Patient-Reported Outcomes

No functional or patient-reported outcome could be synthesised. The instruments prespecified in the protocol were largely absent from this literature: no cohort used the Western Ontario and McMaster Universities Osteoarthritis Index or the SF-12, and the Harris Hip Score was used by a single series and reported as means without dispersion [28]. Two cohorts used the Merle d’Aubigné–Postel score, one with pre-set grade thresholds [1,27]. The registry cohort used no subjective score at all [29]. Because no two cohorts shared an instrument, no continuous outcome could be expressed as a mean difference with a confidence interval as prespecified. No study reported functional outcome stratified by graft-versus-host disease status.

#### 3.5.3. Complications

Surgical and medical complications were reported too inconsistently to synthesise. Only the registry cohort enumerated complications individually, and it is also the only cohort to report a dislocation. Periprosthetic fracture was not reported by any included study. Reoperation could not be separated from revision in any cohort, since no study distinguished reoperations not involving component exchange, so reoperation is subsumed within the all-cause revision estimate reported above rather than counted separately. One series described multiple systemic infections in its cohort but did not enumerate them or relate them to the arthroplasty [28].

#### 3.5.4. All-Cause Mortality

All-cause mortality during follow-up was reported by all four pooled cohorts, though at differing censoring horizons. Sixteen deaths occurred among 135 patients, and pooled mortality was 11.26% (95% CI 5.87–20.54) with substantial heterogeneity (I^2^ = 61%, Q = 7.73, df = 3, *p* = 0.05). That heterogeneity is a function of observation time rather than of case mix: the two cohorts followed longest reported 9 of 61 and 3 of 45 deaths [1,2], the series with complete two-to-eight-year follow-up reported none among 15 patients [27], and the shortest series reported 4 of 14 deaths at a mean of 2.2 years [28]. The last figure is the highest in the review and arises in the cohort with the shortest follow-up and universal chronic graft-versus-host disease, consistent with early transplant-related mortality rather than any arthroplasty-related cause.

Mortality of this magnitude is a competing risk for every implant outcome reported above. Only one included study modelled death as a competing risk, reporting 93% freedom from component revision at five years on that basis [29]. No cohort reported cause of death in a form permitting separation of transplant-related from arthroplasty-related mortality, and none reported mortality by graft-versus-host disease status. Because a patient who dies can no longer be revised, this mortality biases the observed proportions downwards. The survivor-conditional sensitivity analysis in Section 3.4 quantifies the direction and size of that bias: excluding the hips of patients who died raised the revision estimate from 4.39% to 4.98% and the infection estimate from 1.05% to 1.09%.

### 3.6. Implant Survivorship

Survivorship was not pooled because studies reported it at heterogeneous landmarks and against differing endpoints such as component loosening in some and revision in others. The oldest and largest cohort, with a high burden of chronic GVHD, reported a ten-year survivorship of 74.8%, with any-component loosening as the endpoint [1]. The propensity-matched cohort reported revision-free survivorship of 93.4% at 13 years, indistinguishable from idiopathic-osteonecrosis controls (95%, *p* = 0.93), with no infection in either arm [2]. Among the narratively synthesised studies, the registry cohort reported 93% freedom from component revision at five years with death modelled as a competing risk, and noted that revision was not infection-driven [29]. The two shortest series recorded no loosening, infection or revision at means of 56.4 and 44.5 months [27,28]. Apparent improvement across the publication record is consistent with advances in fixation and bearing surfaces, but implant era and GVHD-management era are collinear, so confounding of this trend cannot be excluded.

### 3.7. Graft-Versus-Host Disease as Effect Modifier

No included study reported outcomes with GVHD-stratified denominators, so the prespecified relative-risk meta-analysis and all prespecified subgroup analyses by GVHD type, immunosuppression status, fixation, age, sex and underlying diagnosis were not estimable. The available evidence is nonetheless directionally consistent. In the cohort reporting most failures, all three septic loosenings occurred in patients with severe chronic GVHD on ongoing steroids [1]. In the narrative cohort, both revisions in the transplant group were for suspected infection within the first post-operative year. The matched controls had one revision, and it was not infective. The transplant group was more often immunosuppressed (*p* < 0.001), but GVHD status was not reported [30]. The registry cohort is informative precisely because it is predominantly autologous: 31 of 33 arthroplasties in recipients transplanted for non-leukemic diagnoses were autologous, and these patients cannot develop GVHD. That cohort recorded no infection-driven revision [29]. The remaining pooled cohorts recorded no infection at all [2,27,28]. All three pooled infection events therefore occurred in patients with severe chronic GVHD taking steroids. The narrative cohort suggests the same, but this cannot be confirmed because that study did not record GVHD status for its two revised patients.

### 3.8. Certainty of Evidence

Certainty began at low because every study was non-randomised, and was rated down further for serious risk of bias, driven by unadjusted confounding by GVHD severity and steroid exposure, and for serious to very serious imprecision, reflecting ten revisions and three infection events across 228 hips. Publication bias could not be assessed formally with fewer than ten studies, so certainty was not rated down on that basis. Inconsistency was not a basis for rating down, although with four small studies, the power of I^2^ to detect heterogeneity is limited. Certainty was therefore very low for every outcome (Table 3), and the findings are hypothesis-generating.

## 4. Discussion

### 4.1. Principal Findings

Across four cohorts of exclusively allogeneic recipients and 228 hips, pooled all-cause revision was 4.39% and pooled infection-related failure 1.05%, with little heterogeneity. By the conventional metrics of arthroplasty the reconstruction performs about as well as in the wider osteonecrosis literature, which is not trivial given that these patients reach surgery decades younger than the typical candidate. The more consequential observation is that all three pooled infection events, all of them from a single cohort, occurred in patients with severe chronic GVHD receiving steroids at the time of revision, while mechanical failures showed no such relationship. The pooled figures are best read not as a single reassuring rate but as a low-frequency signal concentrated in a single subgroup.

### 4.2. Interpretation

A coherent mechanism links exposure to outcome. Chronic GVHD and its immunosuppression produce sustained immune impairment, compromised wound healing, mucocutaneous barrier breakdown and a burden of infective episodes, each contributing to periprosthetic seeding [3]. Among osteonecrosis aetiologies, those requiring immunosuppressive medication carry the highest reported risk of joint infection [11]. Case-level evidence supports this: in an earlier series from one included centre, the single deep infection arose in a patient with chronic GVHD two and a half years after surgery, and there was no revision for aseptic loosening [23]. The clustering observed here is what would be predicted if GVHD acts less as a determinant of overall survivorship than as a modifier of the mode of failure, shifting the distribution towards infection without necessarily raising aggregate revision detectably. Notably, a comparably young cohort with corticosteroid-induced osteonecrosis from treatment of haematological malignancy, of similar age and steroid exposure but in which only four of 27 patients had undergone transplantation, required one revision for a fractured ceramic liner and recorded no infection over a mean of 9.3 years [26]. This argues against the idea that steroid exposure alone accounts for the septic signal. These data nonetheless show association, not causation; without stratified denominators, the mechanism remains the most parsimonious reading of a consistent pattern rather than a quantified effect.

### 4.3. Follow-Up and the Zero-Event Cohorts

The most instructive study is the one that argues against the central claim: a cohort in which every patient had chronic GVHD on prolonged high-dose corticosteroids yet no infection or revision occurred, at a mean follow-up of 44.5 months [28]. Septic loosening here is a late event, declaring itself years rather than months after surgery [1,23]. A cohort whose observation ends before that window opens cannot capture the events at issue, so its unblemished record is evidence of short follow-up rather than of absent risk. Follow-up truncation cuts in a second direction. Failure after arthroplasty is not a single hazard: aseptic loosening of early onset declares itself within the first two years, whereas loosening driven by particulate wear debris and the periprosthetic osteolysis it provokes characteristically emerges between five and ten years after implantation. A series followed for a mean of 3.7 to 4.7 years is therefore too short to capture either the septic events at issue here or the wear-related mechanical failures that dominate the second decade, and a null result over that interval speaks to neither. This is the reasoning behind the follow-up-stratified analysis reported in Section 3.4, in which every observed event falls in the stratum followed beyond five years, and it is why the durability reported here should be read as applying to the first post-operative decade rather than to the lifetime of the implant in a population operated on in its fourth decade of life.

### 4.4. Comparison with Existing Literature

Prior syntheses pooled osteonecrosis broadly, or compared it with osteoarthritis, without isolating the transplant subgroup [12,13], and their reassuring aggregate survivorship has understandably been read as reassurance for transplant recipients. The contribution here is to separate that subgroup and show that the aggregate figure, while accurate, is silent on what distinguishes these patients: the shift towards septic failure among the immunosuppressed.

### 4.5. Limitations

The evidence base is small, retrospective, and heterogeneous in how GVHD and immunosuppression are defined and reported. Most decisively, no study reported GVHD-stratified denominators, which precludes a relative-risk estimate and leaves the central signal qualitative. Several further features constrain interpretation. In a young cohort with substantial transplant-related mortality, patients who die can never be revised, and those deaths fall disproportionately among the immunosuppressed patients at highest infection risk. Naïve proportions therefore tend to understate the true infection hazard among survivors. The denominator convention is stated in Section 2.6. In the largest cohort 107 hips were implanted while attrition left 70 reviewed at final follow-up, and the implanted denominator was retained, which makes the resulting proportions conservative. The patient-level totals in that report do not reconcile arithmetically, but the event counts and denominators used here were taken from its hip-level operative and reoperation tables, which are internally consistent, so the discrepancy does not affect the extracted data. Several series report hips rather than patients—the 228 pooled hips came from 135 patients—and two hips in the same recipient share the same immune status and immunosuppressive exposure, so they are not independent observations. The pooled confidence intervals are therefore narrower than the true uncertainty warrants; recomputing them under complete within-patient correlation widens them by a factor of 1.30 while leaving the point estimates unchanged (Section 3.4; Table S6). The endpoints themselves differ across eras, so the temporal trend is confounded by evolving definitions. Furthermore, relative risks, hazard ratios and GVHD subgroup analyses could not be computed without stratified denominators. Funnel plots and Egger’s test were not included as they are unreliable with fewer than ten studies. Aseptic loosening, functional scores and complications were prespecified as secondary outcomes but could not be pooled, because definitions and instruments differed too widely across cohorts for meaningful summary. All-cause mortality was the only secondary outcome that could be synthesised quantitatively. No study reported any secondary outcome by GVHD status. The Freeman–Tukey estimate was demoted to a prespecified sensitivity analysis, and a random-intercept logistic model adopted as the primary synthesis, after its back-transformation substantially understated the observed infection rate, a documented failure at low proportions [20]. The search was restricted to English-language reports, so language bias cannot be excluded, although reference screening identified no eligible non-English report. The review team comprised two reviewers, which meets the PRISMA requirement that selection and extraction be performed independently and in duplicate but leaves no independent arbiter; every disagreement was nonetheless resolved by consensus, and none arose that the primary reports could not settle. MINORS risk of bias assessment replaced the Newcastle–Ottawa scale as it is better suited to non-comparative surgical series. Two studies whose post-allogeneic osteonecrosis subgroup could not be separated were kept as included studies but excluded from the pooled estimates and synthesised narratively [29,30]; the predominantly autologous registry cohort thereby serves as an external comparison rather than diluting the pooled denominator.

### 4.6. Implications and Future Research

These results do not argue for withholding or delaying arthroplasty on the basis of transplant history. The durability is good and the operation remains the definitive treatment for the collapsed osteonecrotic head; joint-preserving surgery performs least well precisely in the corticosteroid-associated disease that characterises this population, steroid-induced osteonecrosis being over-represented among the hips that progress to arthroplasty despite grafting [31]. They argue instead for infection-focused vigilance in the GVHD and immunosuppressed subgroups, informing prevention measures, surveillance for indolent infection, and the threshold for investigating late pain. The single missing ingredient for quantification is stratified reporting. Registry-based and multicentre studies recording GVHD status and grade by NIH consensus criteria [4] and cumulative immunosuppressive exposure alongside arthroplasty outcomes, with competing-risks analysis and follow-ups long enough to capture late septic failure, would resolve the question with adequate power. National arthroplasty registries have already shown that designs of this kind can resolve patient- and implant-level determinants of revision at a resolution no single-centre series can approach [32].

## 5. Conclusions

Total hip arthroplasty for osteonecrosis of the femoral head after allogeneic haematopoietic stem cell transplantation is durable over the first post-operative decade, with pooled all-cause revision of 4.39% and pooled infection-related failure of 1.05%—the latter a lower bound, given the unequal follow-up of the contributing cohorts—and remains the definitive treatment for the collapsed hip in this young population. Beneath that average, the infection-related failures recorded were confined to patients with chronic graft-versus-host disease on continuing immunosuppression—a pattern consistent with the absence of such failures in a predominantly autologous cohort, and with the observation that the series with the cleanest records are those followed for the shortest time. This pattern rests on three events in a single cohort and is therefore hypothesis-generating rather than established. The present evidence can describe this pattern but cannot yet measure it, for want of a single reported variable. Correcting that omission requires no new technology, only that graft-versus-host disease status and immunosuppressive exposure be reported alongside arthroplasty outcomes.

## Figures and Tables

**Figure 1:**
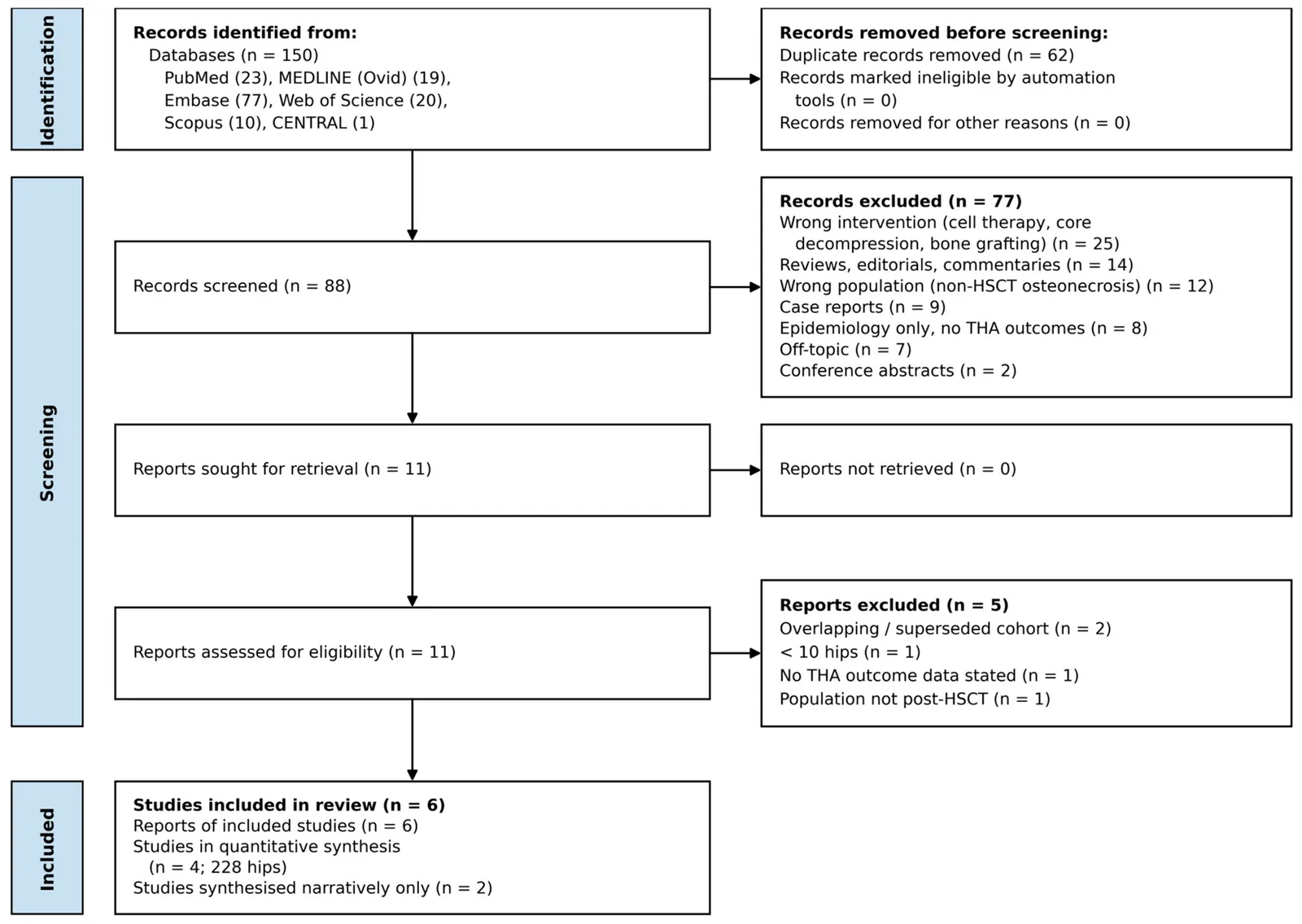
PRISMA 2020 flow diagram for study selection.

**Figure 2:**
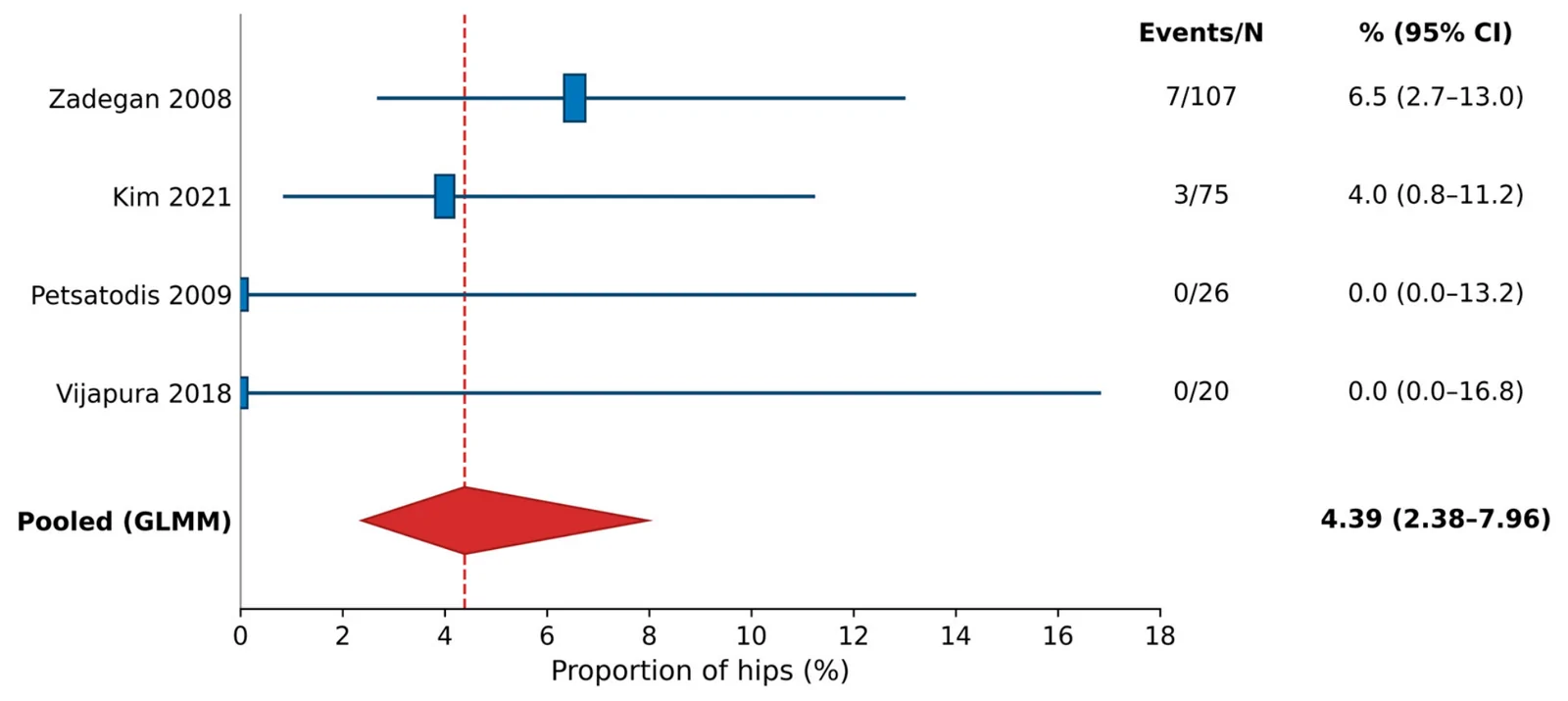
Forest plot of all-cause revision after total hip arthroplasty for osteonecrosis of the femoral head following allogeneic haematopoietic stem cell transplantation. Pooled proportion 4.39% (95% CI 2.38–7.96) from a random-intercept logistic model; I^2^ = 15%, Q = 3.52, df = 3, *p* = 0.32. Study-level intervals are exact binomial and marker area is proportional to cohort size. All four cohorts comprised exclusively allogeneic recipients undergoing arthroplasty for osteonecrosis. Squares mark study-level proportions and horizontal lines their exact 95% confidence intervals; the diamond spans the 95% confidence interval of the pooled estimate, which is marked by the dashed vertical line. Study labels refer to Zadegan et al. [1], Kim et al. [2], Petsatodis et al. [27] and Vijapura et al. [28].

**Figure 3:**
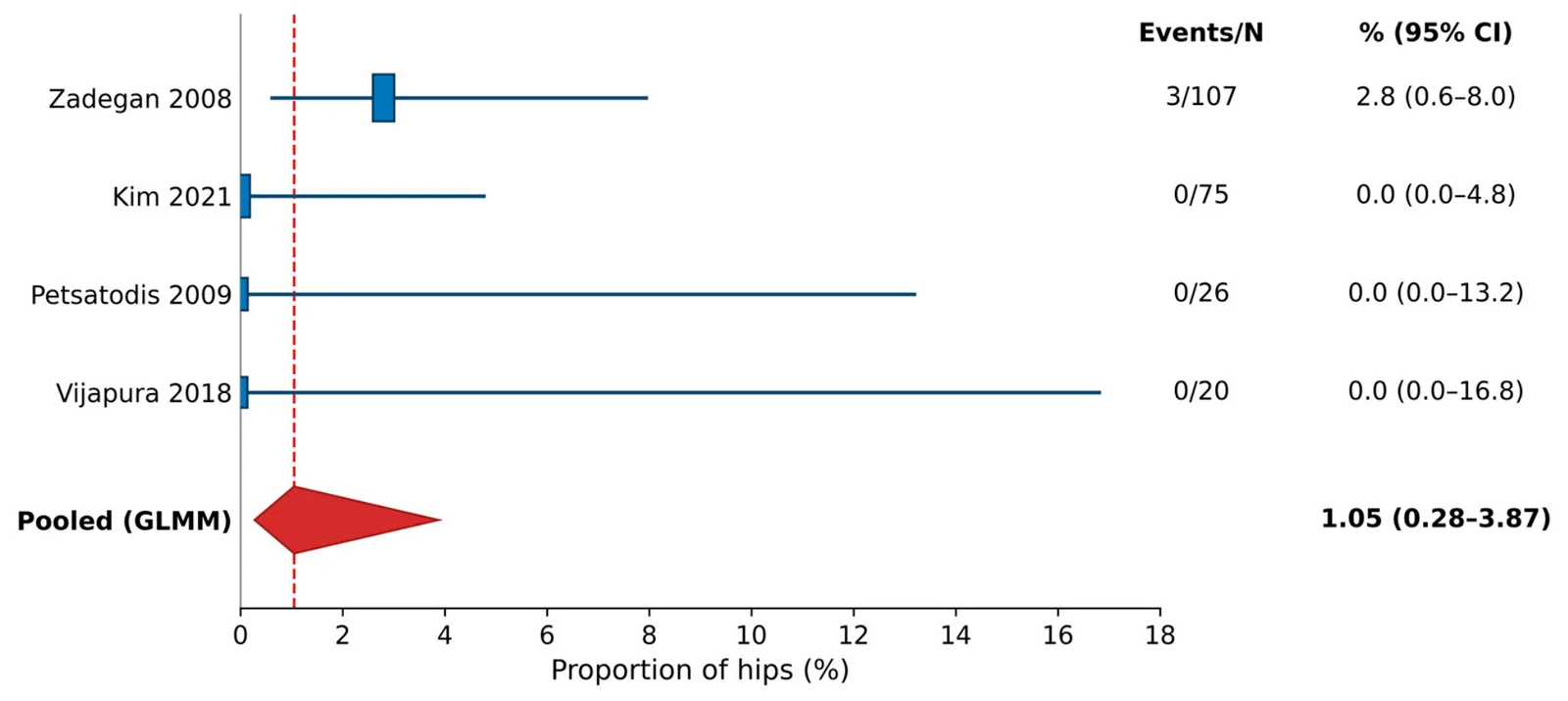
Forest plot of periprosthetic joint infection or septic loosening after total hip arthroplasty for osteonecrosis of the femoral head following allogeneic haematopoietic stem cell transplantation. Pooled proportion 1.05% (95% CI 0.28–3.87) from a random-intercept logistic model; I^2^ = 0%, Q = 2.81, df = 3, *p* = 0.42. Three of four cohorts reported zero events. All three events occurred in a single cohort, in patients with chronic graft-versus-host disease receiving ongoing immunosuppression. Squares mark study-level proportions and horizontal lines their exact 95% confidence intervals; the diamond spans the 95% confidence interval of the pooled estimate, which is marked by the dashed vertical line. Study labels refer to Zadegan et al. [1], Kim et al. [2], Petsatodis et al. [27] and Vijapura et al. [28].

**Table 1:** Characteristics of the six included studies.

Study (Country), Design	Patients/Hips Reported	Hips Analysed	Transplant	GVHD and Immunosuppression	Mean Age, Range	Mean Follow-Up, Range
Zadegan 2008 (France) [1] Retrospective case series	77 patients/123 osteonecrotic hips and 61 patients (107 hips) underwent arthroplasty	107	Allogeneic BMT	62% affected by GVHD. Acute and chronic GVHD on prolonged high-dose steroids. Total body irradiation in all patients.	33 years, (15.7–56) at arthroplasty	9.2 years, (2–26)
Kim 2021 (South Korea) [2] Multicentre propensity-matched cohort	45 patients/75 THAs and separately matched to 75 idiopathic-osteonecrosis control hips not analysed here	75	Allogeneic BMT	32/45 (71%) developed acute and/or chronic GVHD. Calcineurin inhibitor plus systemic steroids noted.	30.7 years at arthroplasty	10.6 ± 3.5 years
Petsatodis 2009 (Greece) [27] Retrospective case series	15 patients/26 consecutive THAs	26	Allogeneic BMT	All patients developed severe prolonged GVHD necessitating steroid therapy (mean 35.2 months).	33.6 years at implantation	56.4 months (24–96)
Vijapura 2018 (USA) [28] Retrospective case series	14 patients/20 THAs and 11/14 (79%) bilateral	20	Allogeneic HSCT	All 14 (100%) had chronic GVHD. Prednisone ~1 mg/kg/day for one to two years. Multiple systemic infections noted.	46 years (26–63) at arthroplasty	44.5 months (9.5–128 months)
Chalmers 2017 (USA) [29] Retrospective single-centre cohort	36 patients/42 arthroplasties (23 osteonecrosis and 19 degenerative)	Not pooled—only used for narrative synthesis	Mixed allogeneic and autologous HSCT: 31/33 arthroplasties for non-leukaemic diagnoses were autologous	GVHD present but not stratified. Immunosuppressive therapy noted.	52 years (25–74) at arthroplasty	5 years (2–15)
Chang 2024 (USA) [30] Retrospective propensity-matched cohort	43 arthroplasty-BMT patients matched to 43 arthroplasty-osteoarthritis controls (hips and knees combined)	Not pooled—only used for narrative synthesis	BMT: allogeneic and autologous not stratified	Post-transplant steroids. Immunosuppression of BMT group noted but GVHD status not specified.	Median 65 years (51–69)	Median 64 months (minimum 2 years)

All were retrospective. The Hips Analysed column gives the hips contributing to the pooled estimates and sums to 228. Chalmers and Chang were synthesised narratively because the post-allogeneic osteonecrosis subgroup could not be separated. BMT—bone marrow transplantation, GVHD—graft-versus-host disease, HSCT—haematopoietic stem cell transplantation, THA—total hip arthroplasty.

**Table 2:** Extracted outcomes and graft-versus-host disease signal.

Study	Hips	All-Cause Revision	PJI/Septic Loosening	Survivorship and GVHD Among Failures
Zadegan 2008 [1]	107	7 (6.5%)	3 (2.8%)	10-year KM 74.8% All three septic loosenings occurred in patients with severe chronic GVHD on steroids at revision
Kim 2021 [2]	75	3 (4.0%)	0 (0%)	13-year KM 93.4% versus 95% in controls, no PJI. 71% had GVHD, revisions not stratified
Petsatodis 2009 [27]	26	0 (0%)	0 (0%)	No loosening or severe complications at mean 56.4 months. Severe prolonged GVHD on steroids in all patients
Vijapura 2018 [28]	20	0 (0%)	0 (0%)	No failures at mean 44.5 months. All patients had chronic GVHD (pure-GVHD cohort)
Chalmers 2017 [29] (narrative)	23 of 42	2 of 42 whole-cohort (not by indication)	0 (0%)	5-year KM 93% with death as competing risk. Both revisions are non-septic (instability, iliopsoas impingement). GVHD not stratified
Chang 2024 [30] (narrative)	Not pooled—43 patients (hips and knees combined)	2 (both for suspected infection)	2	2-year survivorship equal to OA controls. Both revisions were for suspected infection within the first post-operative year. GVHD status not reported per patient
Pooled (4 cohorts)	228	4.39% (95% CI 2.38–7.96)	1.05% (95% CI 0.28–3.87)	I^2^ = 15% revision, 0% infection. All three septic events occurred in chronic-GVHD patients on immunosuppression [1]

Percentages are per hip. Chalmers and Chang are synthesised narratively and do not enter the pooled estimates: Chalmers mixed allogeneic with autologous recipients and reported revisions for the whole cohort rather than by indication, and Chang combined hip with knee arthroplasty and osteonecrosis with osteoarthritis. Chalmers recorded no infection-driven revision, whereas both revisions in Chang’s transplant group were for suspected infection; neither cohort contributes to the pooled estimates. KM Kaplan–Meier, PJI periprosthetic joint infection, CI confidence interval, OA osteoarthritis.

**Table 3:** GRADE summary of findings.

Outcome	Hips (Studies), Follow-Up Range	Pooled Effect (95% CI)	Certainty	Interpretation
All-cause revision	228 (4), 44.5 months–10.6 years	4.39% (2.38–7.96), I^2^ = 15%	⨁◯◯◯ Very low	About 44 per 1000 hips undergo revision. Low, but the true rate is uncertain Restricted to cohorts followed beyond five years, 5.49% (2.98–9.91)
PJI/septic loosening	228 (4), 44.5 months–10.6 years	1.05% (0.28–3.87), I^2^ = 0%	⨁◯◯◯ Very low	Infection-related failure is uncommon. The estimate rests on three events and is fragile, all from one cohort. Restricted to cohorts followed beyond five years, 1.62% (0.51–5.01). The whole-cohort figure is a lower bound
Implant survivorship (Kaplan–Meier)	182 (2), 10–13 years	Not pooled: 74.8% at ten years to 93.4% at 13 years (differing endpoints)	⨁◯◯◯ Very low	Generally durable, but survivorship varies with era, endpoint definition and GVHD burden
Modification of outcome by GVHD/immunosuppression	6 studies with none stratified by GVHD status	Not estimable as there are no GVHD-stratified denominators	⨁◯◯◯ Very low	Septic failures clustered in chronic-GVHD patients on immunosuppression. Direction consistent, magnitude unquantifiable. The co-occurrence is descriptive and rests on three events in a single cohort
All-cause mortality	135 patients (4)	11.26% (5.87–20.54), I^2^ = 61%	⨁◯◯◯ Very low	Substantial competing risk. Censoring horizons differ across cohorts

High (⨁⨁⨁⨁): very confident the true effect is close to the estimate. Moderate (⨁⨁⨁◯). Low (⨁⨁◯◯). Very low (⨁◯◯◯): very little confidence and the true effect is likely to differ substantially. CI—confidence interval, PJI—periprosthetic joint infection, GVHD—graft-versus-host disease.

## Data Availability

The data-extraction table and the analysis code supporting the pooled estimates are available from the corresponding author on reasonable request.

## Supplementary Materials

The following supporting information can be downloaded at: https://www.mdpi.com/article/10.3390/jcm15197528/s1. Table S1: Eligibility criteria (PICOS); Table S2: Full electronic search strategies; Table S3: ROBINS-I domain-level assessments (comparative studies); Table S4: MINORS item-level assessments (non-comparative studies); Table S5: PRISMA 2020 checklist; Table S6: Sensitivity analyses for all-cause revision and for periprosthetic joint infection or septic loosening.
