# Supplementary material for: Total Hip Arthroplasty for Osteonecrosis of the Femoral Head Following Allogeneic Haematopoietic Stem Cell Transplantation, with Graft-Versus-Host Disease as a Candidate Effect Modifier: A Systematic Review and Meta-Analysis
Source: J Clin Med. 2026 Sep 28;15(19):7528. doi: 10.3390/jcm15197528 (PMC13666444; doi:10.3390/jcm15197528)
Supplement: Supplementary file 1 [file jcm-15-07528-s001.zip › jcm-4534682-supplementary.pdf]

Supplementary Materials

Total Hip Arthroplasty for Osteonecrosis of the Femoral Head Following Allogeneic Haematopoietic Stem Cell Transplantation, with Graft-versus-Host Disease as a Candidate Effect Modifier: A Systematic Review and Meta-Analysis

Minki Beak and Young Wook Lim

Table S1. Eligibility criteria (PICOS).

Domain	Criteria
Population	Patients of any age or sex undergoing primary total hip arthroplasty for osteonecrosis or avascular necrosis of the femoral head developing after allogeneic haematopoietic stem cell transplantation. Excluded: osteonecrosis of non-transplant cause (idiopathic, alcohol, trauma, sickle cell, non-transplant steroid use, lupus) unless the post-transplant subgroup was reported separately and patients with osteonecrosis at sites other than the hip.
Intervention / Exposure	Primary total hip arthroplasty using any implant, fixation method or bearing surface. The prespecified effect modifier was graft-versus-host disease (acute and/or chronic) with ongoing corticosteroid or immunosuppressive treatment at or after arthroplasty. GVHD grade and cumulative steroid dose were captured where reported.
Comparator	Patients undergoing arthroplasty for osteonecrosis without graft-versus-host disease or ongoing immunosuppression. Secondarily, non-transplant osteonecrosis or primary osteoarthritis. Single-arm studies without comparators were eligible.
Outcomes	Primary: periprosthetic joint infection or septic loosening; all-cause and cause-specific revision, and Kaplan–Meier implant survivorship. Secondary: aseptic loosening, functional scores (Harris Hip Score, WOMAC, SF-12), complications, mortality.
Study design	Prospective or retrospective comparative cohorts and case series with at least ten hips and a mean follow-up of at least 24 months. Excluded: reviews, editorials, commentaries, case reports, animal studies, and conference abstracts without extractable data.

WOMAC Western Ontario and McMaster Universities Osteoarthritis Index, GVHD graft-versus-host disease

Table S2. Full electronic search strategies

All databases were searched from inception to July 2026 and limited to English. The strategy combined four concept blocks with the Boolean AND operator, and OR within each block. Records retrieved: PubMed 23, MEDLINE (Ovid) 19, Embase 77, Web of Science 20, Scopus 10 and Cochrane CENTRAL 1 (total 150; 62 duplicates removed, leaving 88 for screening). No trial register was searched separately; 2 ClinicalTrials.gov records were retrieved through Embase and are counted within the Embase total.

S2.1. PubMed

(("hematopoietic stem cell transplantation"[MeSH] OR "bone marrow transplantation"[MeSH]
OR "hematopoietic stem cell transplant*"[tiab] OR "haematopoietic stem cell transplant*"[tiab]
OR "bone marrow transplant*"[tiab] OR HSCT[tiab] OR HCT[tiab] OR BMT[tiab] OR allogeneic[tiab])
AND ("graft vs host disease"[MeSH] OR "graft-versus-host"[tiab] OR "graft versus host"[tiab]
OR GVHD[tiab] OR "adrenal cortex hormones"[MeSH] OR "immunosuppressive agents"[MeSH]
OR corticosteroid*[tiab] OR glucocorticoid*[tiab] OR steroid*[tiab] OR immunosuppress*[tiab])
AND ("osteonecrosis"[MeSH] OR "femur head necrosis"[MeSH] OR osteonecrosis[tiab]
OR "avascular necrosis"[tiab] OR "aseptic necrosis"[tiab] OR "ischemic necrosis"[tiab]
OR AVN[tiab] OR ONFH[tiab])
AND ("arthroplasty, replacement, hip"[MeSH] OR "total hip arthroplasty"[tiab]
OR "total hip replacement"[tiab] OR "hip arthroplasty"[tiab] OR "hip replacement"[tiab]
OR THA[tiab] OR THR[tiab]))

S2.2. MEDLINE (Ovid)

1. exp Hematopoietic Stem Cell Transplantation/ or exp Bone Marrow Transplantation/
2. (h?ematopoietic stem cell transplant* or bone marrow transplant* or HSCT or HCT or BMT or allogeneic).ti,ab,kf.
3. 1 or 2
4. exp Graft vs Host Disease/ or exp Adrenal Cortex Hormones/ or exp Immunosuppressive Agents/
5. (graft adj2 host or GVHD or corticosteroid* or glucocorticoid* or steroid* or immunosuppress*).ti,ab,kf.

6. 4 or 5
7. exp Osteonecrosis/ or exp Femur Head Necrosis/
8. (osteonecrosis or avascular necrosis or aseptic necrosis or AVN or ONFH).ti,ab,kf.
9. 7 or 8
10. exp Arthroplasty, Replacement, Hip/
11. (total hip arthroplasty or total hip replacement or hip arthroplasty or hip replacement or THA or THR).ti,ab,kf.
12. 10 or 11
13. 3 and 6 and 9 and 12

S2.3. Embase (Emtree)

1. 'hematopoietic stem cell transplantation'/exp OR 'bone marrow transplantation'/exp
2. (h?ematopoietic stem cell transplant* OR bone marrow transplant* OR HSCT OR HCT OR BMT OR allogeneic):ti,ab,kw
3. 1 OR 2
4. 'graft versus host reaction'/exp OR 'corticosteroid'/exp OR 'immunosuppressive agent'/exp
5. ("graft" NEAR/2 "host" OR GVHD OR corticosteroid* OR glucocorticoid* OR steroid* OR immunosuppress*):ti,ab,kw
6. 4 OR 5
7. 'osteonecrosis'/exp OR 'femur head osteonecrosis'/exp
8. (osteonecrosis OR "avascular necrosis" OR "aseptic necrosis" OR AVN OR ONFH):ti,ab,kw
9. 7 OR 8
10. 'total hip prosthesis'/exp OR 'hip arthroplasty'/exp
11. ("total hip arthroplasty" OR "total hip replacement" OR "hip arthroplasty" OR "hip replacement" OR THA OR THR):ti,ab,kw
12. 10 OR 11
13. 3 AND 6 AND 9 AND 12

S2.4. Cochrane CENTRAL

#1 MeSH descriptor: (Hematopoietic Stem Cell Transplantation) explode all trees
#2 MeSH descriptor: (Bone Marrow Transplantation) explode all trees
#3 ((haematopoietic or hematopoietic or "bone marrow") NEAR/3 transplant*):ti,ab,kw
#4 (HSCT or HCT or BMT or allogeneic):ti,ab,kw
#5 #1 OR #2 OR #3 OR #4
#6 MeSH descriptor: (Graft vs Host Disease) explode all trees
#7 (GVHD or "graft versus host" or corticosteroid* or glucocorticoid* or steroid* or immunosuppress*):ti,ab,kw
#8 #6 OR #7
#9 MeSH descriptor: (Osteonecrosis) explode all trees
#10 (osteonecrosis or "avascular necrosis" or "aseptic necrosis" or AVN or ONFH):ti,ab,kw
#11 #9 OR #10
#12 MeSH descriptor: (Arthroplasty, Replacement, Hip) explode all trees
#13 (("total hip" NEAR/2 (arthroplasty or replacement)) or THA or THR):ti,ab,kw
#14 #12 OR #13
#15 #5 AND #8 AND #11 AND #14

S2.5. Web of Science Core Collection

TS=(("hematopoietic stem cell transplant*" OR "haematopoietic stem cell transplant*" OR "bone marrow transplant*" OR HSCT OR HCT OR BMT OR allogeneic)
AND ("graft-versus-host" OR "graft versus host" OR GVHD OR corticosteroid* OR glucocorticoid* OR steroid* OR immunosuppress*)
AND (osteonecrosis OR "avascular necrosis" OR "aseptic necrosis" OR AVN OR ONFH)
AND ("total hip arthroplasty" OR "total hip replacement" OR "hip arthroplasty" OR "hip replacement" OR THA OR THR))

S2.6. Scopus

TITLE-ABS-KEY(("hematopoietic stem cell transplant*" OR "haematopoietic stem cell transplant*" OR "bone marrow transplant*" OR HSCT OR HCT OR BMT OR allogeneic)

AND ("graft-versus-host" OR "graft versus host" OR GVHD OR corticosteroid*
OR glucocorticoid* OR steroid* OR immunosuppress*)
AND (osteonecrosis OR "avascular necrosis" OR "aseptic necrosis" OR AVN OR ONFH)
AND ("total hip arthroplasty" OR "total hip replacement" OR "hip arthroplasty"
OR "hip replacement" OR THA OR THR))

Table S3. ROBINS-I domain-level assessments (comparative studies).

Domain	Kim 2021 [2]	Chang 2024 [30]
Confounding	Serious – propensity matching on age, sex, BMI, ASA and year of surgery. GVHD status/grade and cumulative steroid dose neither matched nor adjusted, although 71% had GVHD	Serious – 1:1 matching on surgical site, sex, age, BMI, ASA and Elixhauser index only, without propensity scores or adjusted modelling; transplant type is never specified as allogeneic and GVHD was not recorded; the comparator is primary osteoarthritis rather than osteonecrosis, and a ten-year age difference persists after matching (47 vs 57 years, P = .055)
Selection of participants	Moderate – all arthroplasties for osteonecrosis after BMT at two institutions (1997–2012) with ≥5-year follow-up; retrospective but institution-wide	Serious – single-centre database review (2013–2021) requiring ≥ 2-year follow-up. No screening, exclusion or attrition counts are given, so the extent of selection on post-operative survival cannot be judged. The authors state that they did not examine how patients were selected for surgery
Classification of interventions	Low – arthroplasty recorded contemporaneously in operative records	Moderate – arthroplasty and transplant history recorded contemporaneously in the institutional database, but osteonecrosis was inferred from radiographs rather than confirmed on MRI in most patients, and two of 36 hip recipients were transplanted for non-haematological disease
Deviations from intended interventions	Low – single surgical intervention by two senior surgeons, standardised posterolateral approach	Low – single surgical intervention performed by fellowship-trained arthroplasty surgeons within one adult reconstruction division
Missing data	Low – five patients lost with osseointegrated components and no revision at last contact. Three (four hips) died before five years; attrition documented and minimal for the outcome	Moderate – no participant flow or loss to follow-up is reported; cause of death is unknown in five of eight deaths; survivorship is given as crude percentages without Kaplan–Meier estimation or a censoring model
Measurement of outcomes	Low – deep infection by Musculoskeletal Infection Society criteria; radiographs read by two independent observers not involved in surgery	Moderate – revision and return to theatre captured from institutional records, but no case definition is given for periprosthetic joint infection (reported as revision for "PJI concern"); single-centre capture may miss revisions performed elsewhere, and transplant recipients are followed more intensively than the comparator
Selection of the reported result	Moderate – prespecified outcome list stated. No prospective protocol registration	Serious – no prospective protocol registration. The two-year analysis window was chosen post hoc "to maintain reasonable sample sizes" despite a median follow-up of 64 months, and roughly forty unadjusted comparisons are reported across two joints
OVERALL	Serious – taken from the least favourable domain (confounding). Every other domain is low or moderate, so this is otherwise the strongest study in the review	Serious – taken from confounding, selection and reporting. An unverifiable transplant type, a presumptive osteonecrosis diagnosis and a two-year horizon mean the study contributes short-term proportions only and no information on GVHD

ROBINS-I judgments are result-specific. The result assessed is implant survivorship, all-cause revision and periprosthetic joint infection or septic loosening after total hip arthroplasty, and whether GVHD modifies these outcomes. The overall judgment for each study equals the least favourable domain judgment.

BMI body mass index, ASA American Society of Anesthesiologists, BMT bone marrow transplantation, GVHD graft-versus-host disease. MRI magnetic resonance imaging, PJI periprosthetic joint infection.

Neither study reports GVHD-stratified implant outcomes. Kim et al. record GVHD status (32 of 45 patients affected) but present no stratified analysis. Chang et al. did not ascertain GVHD. GVHD as an effect modifier is therefore reported narratively rather than pooled

Table S4. MINORS item-level assessments (non-comparative studies).

MINORS item	Zadegan 2008	Petsatodis 2009	Chalmers 2017	Vijapura 2018
1. A clearly stated aim	2 - Clear objectives: whether THA improved functional status, led to a high rate of complications	2 - Explicit hypothesis with population, intervention and a pre-set minimum: to present the	2 - Clearly quantifiable objectives: overall mortality, complication rates, and revision,	1 - Well-stated objective: "To evaluate outcomes in a series of HSCT recipients who

MINORS item	Zadegan 2008	Petsatodis 2009	Chalmers 2017	Vijapura 2018
	(loosening, osteolysis, sepsis), and gave an acceptable 10-year survival rate.	results of 26 cementless THAs in 15 patients "with a minimum follow-up of 2 years and to examine the hypothesis that cementless techniques can lead to encouraging results."	reoperation and implant survivorship.	underwent THA for AVN." Population and intervention are precise, but no primary outcome, hypothesis or time horizon is nominated.
2. Inclusion of consecutive patients	1 - All-comers departmental series, January 1980 to October 2004, but the word "consecutive" never appears and no screening algorithm is described. Exclusion of the 16 medically treated patients is by indication, not selection.	2 - Explicitly stated: "From January 1998 until December 2004, we performed 26 consecutive cementless total hip arthroplasties in 15 patients." Defined period, single unit.	2 - Exhaustive dual-database capture: transplant diagnosis codes searched, then "cross-referenced that patient population with the institution's total joint registry", with explicit age and procedure eligibility criteria. Vetting for surgery was intensive, but that is a generalisability issue, not incomplete study capture.	1 - "14 HSCT patients who underwent 20 THAs between 2006 and 2014 were retrospectively reviewed." Defined period and single practice, but consecutiveness not stated and no source denominator, screening or exclusion counts given.
3. Prospective collection of data	1 - Explicitly retrospective across a 24-year span, with no protocol predating collection and no registry. Not 0 because the method is clearly stated and validated instruments were applied.	1 - Neither "retrospective" nor "prospective" appears and no protocol is described. A standardised baseline in every patient and a fixed schedule (weeks 6, 12, 24, then annually) are consistent with prospective collection but are not stated as such.	1 - Explicitly stated to be retrospective so capped at 1. The strongest 1 of the four: outcomes were drawn partly from a prospectively maintained institutional total joint registry.	1 - Explicitly retrospective, but data sources are enumerated rather than vague (multidisciplinary notes, operative reports, discharge summaries, clinical examinations).
4. Endpoints appropriate to the aim of the study	2 - Each aim matched and instrument-anchored: Merle d'Aubigné–Postel, Devane activity and Sedel global grading. Loosening defined quantitatively (cup migration >3 mm, rotation >3°, radiolucency >2 mm). Osteolysis defined and actuarial survivorship with 95% CI.	2 - Merle d'Aubigné–Postel with pre-stated grade thresholds (18–17 excellent, 16 good, 15–14 fair, ≤13 poor). Radiographic assessment by Engh criteria with DeLee–Charnley and Gruen zones. Statistical plan pre-stated. No revision or survivorship endpoint.	2 - Every question quantified: Kaplan–Meier patient survivorship at 1, 2 and 5 years. Four complications enumerated individually. Revision counts plus Kaplan–Meier and a competing-risk analysis with death as the competing event. Deep PJI reported discretely (0/42).	1 - Endpoints listed but not defined: no loosening threshold, no radiographic zone system, no wear measurement method, no PJI case definition and no definition of what constituted a complication. Harris hip score reported as means only.
5. Unbiased assessment of the study endpoints	1 - Grading was by two named authors (FZ, AR) who were not the operating surgeons (LS, RN) - a genuine safeguard. But no blinding, no duplicate reading, no inter-observer agreement, and the primary functional endpoints are subjective.	1 - Patients were "reviewed independently by the senior author," who belongs to the operating team. This is independence from other assessors, not from the intervention. Radiographic criteria are objective, but there is no blinding or duplicate reading.	2 - Endpoints assessed are hard and objective (death, revision, component removal, reoperation), registry-captured, and abstracted "by a researcher (BPC) not involved in the initial surgery." No subjective score is used anywhere. Limitations: single abstractor, no PJI case definition.	0 - No independent or blinded assessment reported in any form. Outcomes captured at the treating surgeons' own clinic, no radiographic reader identified, and the global outcome is patient self-report: "Patients subjectively self-reported their overall outcome as good to excellent in all hips."
6. Follow-up period appropriate to the aim of the study	2 - Minimum 2 years, mean 9.2 years (range 2–26). Long enough to capture the events of interest: septic loosening occurred at 6.5, 7 and 9.4 years, and aseptic revisions at 5 to 14.5 years.	2 - 2 to 8 years, mean 56.5 months. The 2-year minimum specified in the aim was met in every patient. Short for wear, which the authors did not measure.	1 - Mean 5 years (range 2–15) but heavily front-loaded: 13 of 37 THAs (31%) analysed at 2 years. Cannot reach the late septic failure window the authors themselves identify as the principal hazard (>5 years in Zadegan et al.)/	1 - Mean 44.5 months, minimum 9.5 months - too short to support the wear, osteolysis and loosening endpoints the study nominates. Conceded by the authors as "short-term follow-up."
7. Loss to follow-up less than 5%	1 - Above 5% but enumerated: 9 patients (15 hips) died, 2 (3 hips) were untraceable, 4 (6 hips) were lost and 7 (13	2 - Unambiguous and complete: "None of the 15 patients was lost or died within the follow-up period." Zero attrition,	1 - Precisely quantified but marginally over threshold: "two of 36 patients (6%) had not been seen in the last 5	1 - Loss to follow-up never stated as such. Four of 14 patients (29%) died at a mean of 2.2 years, accounted for by number,

MINORS item	Zadegan 2008	Petsatodis 2009	Chalmers 2017	Vijapura 2018
	hips) had under 2 years, leaving 49 patients (70 hips). True loss 9/107 hips (8.4%) with total attrition 37/107 (34.6%). Patient-level arithmetic does not reconcile (61 - 22 = 39, not 49).	explicitly stated - the only study of the four to meet the threshold.	years and so are considered lost to followup." 3 patients (8%) died before 2 years. All five were retained: "These five patients (5 THAs) were included in survivorship analyses and calculation of complications."	timing and cause. With that mortality and a 9.5-month minimum, effective attrition for implant endpoints far exceeds 5%.
8. Prospective calculation of the study size	0 - Absent. Actuarial survivorship with 95% CIs is reported, but no a priori power or precision calculation.	0 - Absent. Tests and software specified (SPSS 13, Wilcoxon signed-rank) but no a priori sizing.	0 - Absent. An alpha is declared but no power calculation. The authors concede "a relatively small number of patients."	0 = Absent. Fourteen patients and 20 hips, with no power justification and no inferential statistics attempted.
**TOTAL SCORE (maximum 16)**	**10 / 16**	**12 / 16**	**11 / 16**	**6 / 16**
**OVERALL RISK OF BIAS**	**Moderate risk of bias**	**Moderate risk of bias**	**Moderate risk of bias**	**High risk of bias**

Scoring (MINORS, non-comparative version): 0 = not reported; 1 = reported but inadequate; 2 = reported and adequate. Maximum attainable score 16. Risk of bias categories: 0–4 very high. 5–8 high. 9–12 moderate. 13–16 low.

No study performed a prospective sample size calculation (item 8 scored 0 throughout), which caps the attainable score at 14 in this literature and means every reported null result and zero-event proportion is unpowered.

MINORS measures methodological reporting quality, not evidential weight for the review question. Petsatodis et al. score highest (12/16) on the strength of explicit consecutiveness, a stated hypothesis and zero attrition, but the study includes 15 patients and reports no survivorship or revision endpoint. Chalmers et al. score 11/16 yet provide the only Kaplan–Meier survivorship with competing-risk adjustment for death and the only explicitly independent outcome assessor. The ranking should not be read as a hierarchy of evidence.

Follow-up duration and the infection outcome are structurally mismatched in three of the four series assessed here. Zadegan et al. is the only series whose follow-up reaches the interval in which septic loosening occurred (6.5–9.4 years) and the only one to observe any; the zero deep-infection counts reported by Petsatodis et al. (mean 4.7 years), Chalmers et al. (mean 5 years) and Vijapura et al. (mean 3.7 years) all derive from follow-up shorter than that interval. Of these three, only Petsatodis et al. and Vijapura et al. contribute to the pooled infection estimate, Chalmers et al. being synthesised narratively; the remaining pooled cohort, Kim et al., reaches the relevant interval at a mean of 10.6 years and also recorded no infection. The pooled periprosthetic joint infection proportion is therefore liable to downward bias from follow-up truncation in two of its four contributing cohorts rather than from true differences in risk. A follow-up-stratified sensitivity analysis was therefore performed at revision and is reported in Table S6 and in Section 3.4: every observed event falls in the stratum followed beyond five years, and the two cohorts followed for means of 3.7 and 4.7 years contributed no event in 46 hips.

AVN, avascular necrosis - CI, confidence interval - HSCT, haematopoietic stem cell transplantation - PJI, periprosthetic joint infection - THA, total hip arthroplasty

Table S5. PRISMA 2020 checklist.

Item	Checklist item	Reported / how addressed	Location
**TITLE**			
1	Title	Identifies the report as a systematic review	Title
**ABSTRACT**			
2	Abstract	Structured abstract reporting purpose, methods, results and conclusion	Abstract
**INTRODUCTION**			
3	Rationale	Describes the rationale in the context of existing knowledge: prior syntheses pooled osteonecrosis broadly without isolating the transplant subgroup, and none treated GVHD as the explanatory variable	Section 1
4	Objectives	Explicit objectives: implant survivorship, all-cause revision and periprosthetic joint infection or septic loosening, and whether GVHD modifies these outcomes	Section 1 (final paragraph); Abstract
**METHODS**			
5	Eligibility criteria	Inclusion and exclusion criteria and how studies were grouped	Section 2.2; full PICOS in Table S1

Item	Checklist item	Reported / how addressed	Location
6	Information sources	All databases named with the date each was last searched; no trial register was searched separately; reference lists of included studies were screened	Section 2.3; Table S2
7	Search strategy	Full search syntax reproduced for all six databases, including all filters and limits applied	Table S2
8	Selection process	Two reviewers screened titles, abstracts and full texts independently; disagreements resolved by discussion until consensus, with no third-party adjudication required; records de-duplicated in reference-management software	Section 2.4
9	Data collection process	Two reviewers extracted data independently; every report read in full; denominators and event counts taken from operative and reoperation tables in preference to summary statements	Section 2.4
10a	Data items (outcomes)	All outcomes sought and the methods used to decide which results to collect. Primary: periprosthetic joint infection or septic loosening, revision, Kaplan–Meier survivorship. Secondary: aseptic loosening, functional scores, complications and mortality; of these only all-cause mortality could be synthesised quantitatively, as declared in Limitations	Section 2.2; Table S1
10b	Data items (other variables)	Study, population, transplant, GVHD and immunosuppression, implant and follow-up variables; assumptions about missing data are stated where bounding was used	Section 2; Table 1; Table 2
11	Study risk of bias assessment	ROBINS-I across seven domains for comparative studies (overall judgement from the least favourable domain); MINORS scored 0–2 over eight items (maximum 16) for non-comparative series; two reviewers, independently	Section 2.5; Tables S3 and S4
12	Effect measures	Proportions of hips with 95% confidence intervals, with cluster-robust intervals reported as a sensitivity analysis because 228 hips came from 135 patients. Relative risks were prespecified but were not estimable because no study reported GVHD-stratified denominators	Section 2.6; Section 3
13a	Synthesis: eligibility for synthesis	Four cohorts of exclusively allogeneic recipients undergoing arthroplasty for osteonecrosis entered the quantitative synthesis; two studies whose post-allogeneic osteonecrosis subgroup could not be separated were synthesised narratively	Section 3.1; Figure 1
13b	Synthesis: data preparation	Two studies with mixed populations were excluded from the pooled estimates; no imputation or bounding analysis was required for the four pooled cohorts	Section 2; Section 3; Table 2 footnote
13c	Synthesis: tabulation and visual display	Study-level results tabulated and displayed as forest plots with exact study-level intervals	Table 2; Figures 2 and 3
13d	Synthesis: methods and rationale	Proportions pooled with a random-intercept logistic model (binomial–normal generalised linear mixed model) fitted by adaptive Gauss–Hermite quadrature, which models the binomial likelihood directly, requires no continuity correction and admits zero-event cohorts. The registered DerSimonian–Laird random-effects model on Freeman–Tukey double-arcsine transformed data was retained as a prespecified sensitivity analysis but demoted from the primary analysis because the inverse transformation substantially understated the observed infection rate. Exact (Clopper–Pearson) intervals on the aggregated counts are reported as a second, purely descriptive sensitivity analysis. Survivorship was not pooled because studies reported it at differing landmarks against differing endpoints	Section 2.6
13e	Synthesis: heterogeneity	Between-study heterogeneity summarised by τ^2 from the mixed model and, because Q and I^2 are scale-dependent, by Cochran's Q and I^2 computed on the double-arcsine scale. Subgroup analyses by GVHD type, immunosuppression status, fixation, age, sex and diagnosis were prespecified but were not estimable for want of stratified denominators	Section 2.6; Section 3

Item	Checklist item	Reported / how addressed	Location
13f	Synthesis: sensitivity analyses	Five sensitivity analyses are reported alongside the primary random-intercept logistic model: the prespecified Freeman–Tukey double-arcsine model, exact (Clopper–Pearson) intervals on the aggregated counts, stratification by mean follow-up above and below five years, cluster-robust intervals under a design effect of 1.689, and a survivor-conditional analysis excluding the hips of patients who died. All are tabulated together in Table S6	Section 2.6; Section 3.4
14	Reporting bias assessment	Funnel plots and Egger's test were not performed because fewer than ten studies were available; both are underpowered and unreliable at this number. This is stated as a limitation rather than left implicit	Section 2; Section 4.5
15	Certainty assessment	GRADE applied to each outcome; certainty began at low because every study was non-randomised and was rated down further for serious risk of bias and for serious to very serious imprecision	Section 2; Section 3.8; Table 3
**RESULTS**			
16a	Study selection	150 records identified; 62 duplicates removed; 88 screened; 77 excluded; 11 full texts assessed; six studies included, four pooled and two narrative	Section 3; Figure 1
16b	Studies excluded	All five reports excluded at full-text review are named in the text with their reasons and cited individually: two superseded overlapping cohorts [23, 24], one below the ten-hip minimum [25], one without arthroplasty outcomes [7], and one in a non-transplant population [26]	Section 3.1; Figure 1; references [7], [23], [24], [25], [26]
17	Study characteristics	Country, design, patients and hips, transplant type, GVHD and immunosuppression, age and follow-up for each study	Table 1; Section 3
18	Risk of bias in studies	Domain-level ROBINS-I judgements and item-level MINORS scores presented for every included study	Tables S3 and S4; Section 3
19	Results of individual studies	Hips, all-cause revision, infection events and survivorship reported for each study with exact confidence intervals	Table 2; Figures 2 and 3
20a	Syntheses: characteristics	Four cohorts, 228 hips from 135 patients; contributing studies identified for each synthesis	Section 3; Table 2
20b	Syntheses: results	Pooled all-cause revision 4.39% (95% CI 2.38–7.96); pooled periprosthetic joint infection or septic loosening 1.05% (95% CI 0.28–3.87), with Q and P values reported	Section 3.4; Figures 2 and 3
20c	Syntheses: heterogeneity	$\tau^2 < 0.001$ on the logit scale for revision and $\tau^2 = 0.30$ on the logit scale for infection. On the double-arcsine scale, Q = 3.52, df = 3, P = 0.32, I^2 = 15% (revision) and Q = 2.81, df = 3, P = 0.42, I^2 = 0% (infection). The limited power of I^2 with four small studies is acknowledged, and the between-study variance for infection is poorly identified because three of the four cohorts reported no events	Section 3.4
20d	Syntheses: sensitivity analyses	The Freeman–Tukey model gave 3.20% (95% CI 0.79–6.70) for revision and a point estimate of 0.49% for infection; exact binomial intervals on the aggregated counts gave 4.39% (95% CI 2.12–7.92) and 1.32% (95% CI 0.27–3.80). Restricted to the two cohorts followed beyond five years, revision was 5.49% (95% CI 2.98–9.91) and infection 1.62% (95% CI 0.51–5.01), the two shorter cohorts contributing no event in 46 hips. Cluster-robust intervals were 1.97–9.46 and 0.19–5.66 with point estimates unchanged, and survivor-conditional estimates were 4.98% (95% CI 2.70–9.00) and 1.09% (95% CI 0.26–4.42). Table S6	Section 3.4
21	Reporting biases	Not formally assessed; fewer than ten studies. Declared, not omitted silently	Section 2; Section 4.5
22	Certainty of evidence	Certainty rated very low for every outcome, with the reasons for each downgrade given	Section 3; Table 3
**DISCUSSION**			
23a	Interpretation	Results interpreted in the context of the mechanism linking chronic GVHD and immunosuppression to periprosthetic seeding, and against the wider osteonecrosis literature	Sections 4.1 and 4.2

Item	Checklist item	Reported / how addressed	Location
23b	Limitations of the evidence	Small, retrospective and heterogeneous evidence base; no GVHD-stratified denominators; revision is a competing-risks endpoint; hips rather than patients are reported, so observations are not independent; endpoint definitions differ across eras	Section 4.5
23c	Limitations of the review processes	Deviations from the registered protocol are declared in full, including the change of primary estimator to a random-intercept logistic model, the substitution of MINORS for the Newcastle–Ottawa scale, the retention of one cohort mixing allogeneic with autologous recipients, and the analyses that could not be performed. The search was restricted to English-language reports, so language bias cannot be excluded	Section 4.5
23d	Implications	Implications for practice, policy and future research, with the specific reporting change needed to quantify the effect	Section 4.6; Section 5
**OTHER INFORMATION**			
24a	Registration	Registered with PROSPERO, CRD420261446131, before formal screening began	Section 2.1; Front matter
24b	Protocol access	The registered protocol is publicly available in the PROSPERO record	Section 2; Front matter
24c	Amendments	All deviations from the registered protocol are reported and each is given a reason	Section 4.5
25	Support	No external funding was received	Funding
26	Competing interests	The authors declare no competing interests	Conflicts of Interest
27	Availability of data, code and other materials	The data-extraction table and analysis code are available from the corresponding author on reasonable request; full search syntax is supplied in Supplementary Table S2	Data Availability Statement; Table S2

Items 14 and 21 record that reporting-bias assessment was not undertaken. With six included studies, funnel-plot asymmetry and Egger's test are uninformative, and PRISMA asks that the omission be stated rather than concealed. Item 12 records that the prespecified relative-risk analysis was not estimable; this is reported as a principal finding of the review rather than as an omission, because the absence of GVHD-stratified denominators is the evidence gap the review was designed to test.

Table S6. Sensitivity analyses for all-cause revision and for periprosthetic joint infection or septic loosening.

Analysis	Cohorts (hips)	All-cause revision, % (95% CI)	PJI / septic loosening, % (95% CI)
Primary: random-intercept logistic model	4 (228)	4.39 (2.38–7.96)	1.05 (0.28–3.87)
Freeman–Tukey double arcsine (registered)	4 (228)	3.20 (0.79–6.70)	0.49 (point estimate)
Exact binomial on aggregated counts	4 (228)	4.39 (2.12–7.92)	1.32 (0.27–3.80)
Mean follow-up ≥ 5 years	2 (182)	5.49 (2.98–9.91)	1.62 (0.51–5.01)
Mean follow-up < 5 years	2 (46)	0.00 (0.00–7.71)	0.00 (0.00–7.71)
Cluster-robust, $\varrho = 1$ (design effect 1.689)	4 (228)	4.39 (1.97–9.46)	1.05 (0.19–5.66)
Survivor-conditional denominator	4 (201)	4.98 (2.70–9.00)	1.09 (0.26–4.42)

All estimates are proportions of hips. The primary, cluster-robust and survivor-conditional rows and the two follow-up strata are from the random-intercept logistic model; the exact rows are Clopper–Pearson intervals on the aggregated counts. The ≥ 5-year stratum comprises Zadegan et al. (mean 9.2 years) and Kim et al. (mean 10.6 years); the < 5-year stratum comprises Vijapura et al. (mean 3.7 years) and Petsatodis et al. (mean 4.7 years), which together recorded no event of either kind, so the exact interval is given. The cluster-robust row multiplies the variance of the pooled logit by a design effect of $1 + (\bar{m} - 1)\varrho$, with $\bar{m} = 228/135 = 1.689$ hips per patient and $\varrho = 1$, the worst case of complete within-patient correlation; point estimates are unaffected. The survivor-conditional row removes the 27 hips of the 16 patients who died during follow-up. CI, confidence interval; PJI, periprosthetic joint infection.
